# An in vivo confocal microscopy study of corneal changes in patients with systemic sclerosis

**DOI:** 10.1038/s41598-021-90594-9

**Published:** 2021-05-27

**Authors:** Eszter Szalai, Gabriella Szucs, Szilvia Szamosi, Zsuzsa Aszalos, Ildiko Afra, Adam Kemeny-Beke

**Affiliations:** 1 Department of Ophthalmology, University of Pecs, Rakoczi u. 2, 7623 Pecs, Hungary; 2 Department of Ophthalmology, Faculty of Medicine, University of Debrecen, Nagyerdei krt. 98, 4032 Debrecen, Hungary; 3 Department of Rheumatology, Institute of Medicine, Faculty of Medicine, University of Debrecen, Nagyerdei krt. 98, 4032 Debrecen, Hungary; 4 Department of Immunology, Institute of Medicine, Faculty of Medicine, University of Debrecen, Moricz Zsigmond krt. 22, 4032 Debrecen, Hungary

**Keywords:** Rheumatology, Musculoskeletal system, Rheumatic diseases

## Abstract

To investigate corneal microstructure of systemic sclerosis (SSc) patients using in vivo confocal microscopy (IVCM). 33 patients with SSc and 30 age-matched healthy subjects were recruited. All participants underwent comprehensive ophthalmic examination including IVCM (Heidelberg Retina Tomograph III, Heidelberg Engineering GmbH, Heidelberg, Germany) and ocular surface evaluation. Subbasal nerve plexus morphology was investigated using automated software analysis (ACCMetrics V3; University of Manchester, Manchester, UK). Keratocyte cell densities in the anterior stroma were significantly lower in patients with SSc compared to controls (P < 0.0001). In 7 SSc patients no keratocyte nuclei were identified in the anterior stroma and in most patients scattered hyperreflective punctate material were observed in the anterior stroma. Significantly lower subbasal nerve fiber parameters were found in patients with SSc compared to healthy subjects (P < 0.05). There were no significant correlations between the duration of SSc and any of the corneal cell density values. Tear break-up time values (4.82 ± 3.15 s) and Ocular Surface Disease Index scores (33.27 ± 30.11) were abnormal, Schirmer values (6.78 ± 5.82 mm) were borderline in SSc patients. In SSc, corneal morphological changes and accumulation of punctate material in the stroma was detected with confocal microscopy. Severe ocular surface disease was observed in SSc patients with significant impairment in subbasal nerve plexus morphology resembling peripheral neuropathy.

## Introduction

Systemic sclerosis or scleroderma (SSc) is a complex, chronic autoimmune disease of unknown origin. It is classified as a connective tissue disorder. In addition to pathologic changes in the skin and joints, multiple visceral organs can be affected^1^. The clinical and pathological features of SSc are three separate processes: (1) generalized small vessel vasculopathy, (2) dysregulation of both the innate and adaptive immune system, and (3) dysfunction of fibroblast synthesis^2^. SSc patients are categorized into diffuse cutaneous (dcSSc), limited cutaneous (lcSSc), and sine scleroderma (ssSSc) subsets^3^.

Being a multiple organ disease SSc has a wide range of manifestations, including the eyes. Ocular involvement in SSc is not uncommon and includes changes in the skin of the eyelids, anterior and posterior segments, and the orbit^4–7^. Ocular changes are often first diagnosed in the latter stages of disease, since eye findings typically go unnoticed early and are eclipsed by the skin and internal organ manifestations.

The cornea has definite morphological and immunological attributes, including the occurrence of Langerhans cells (antigen-presenting immune cells), the surrounding blood vessels of the conjunctiva and episcleral layer, the rich content of diverse high-molecular-weight molecules and immune complex depositions, such as immunoglobulin M (IgM) and complement C1 that make it susceptible to immune-related diseases^8^. Numerous corneal parameters, including curvature, pachymetry data, and corneal volume can be altered^9^ and vision threatening complications can also occur during the disease^10^, corneal findings in SSc are underrepresented in the literature.

In vivo confocal microscopy (IVCM) is a helpful tool in the investigation of the corneal structure of SSc patients and can provide high resolution, wide depth-of-focus, and steady-state evaluation of the ocular surface at the cellular level^11^. The aim of this study was to evaluate ocular surface and corneal ultrastructural changes including morphology of corneal subbasal nerve fibers in detail using in vivo confocal microscopy and to compare the data with those of healthy controls. Although corneal alterations have previously been investigated, to the best of our knowledge, no study has evaluated these impairments in the context of corneal IVCM characteristics.

## Patients and methods

### Features of patients

Consecutive SSc patients diagnosed according to the corresponding international criteria^12^ were recruited into our prospective, cross-sectional, observational, case–control study, from the outpatient clinic of the Department of Rheumatology of the University of Debrecen.

### Features of controls

Control subjects were selected from patients presenting for routine eye examination with minor refractive errors (± 1.0 diopter), without any history of systemic diseases such as diabetes mellitus, rheumatic diseases, SS, connective tissue disorders (Weill–Marchesani syndrome, Ehlers-Danlos syndrome, osteogenesis imperfecta, Marfan syndrome, etc.). Eye drop administration within two weeks prior to measurements was also an exclusion criterion in both the patient group and the control group, and no eye drops were allowed during study days. Other exclusion criteria were abnormal eyelid position and closure, contact lens wearing, history of ocular surgery, presence of ocular infection, inflammation of sclera, episcleral layer or uvea, trauma of the eye, peripheral or central corneal melting.

### Ocular and clinical examinations

All participants underwent a comprehensive ophthalmological evaluation, including noncycloplegic best spectacle-corrected visual acuity (BCVA) in decimal chart, intraocular pressure (IOP) measurement, and broad beam examination of the slit lamp to determine the condition of the ocular surface and surrounding tissues, as well as to observe eyelids, conjunctival changes, corneal impairments, and tear film.

Tear break-up time (BUT) followed by Schirmer-I testing was performed for dry eye disease (DED) evaluation. For BUT measurement a strip of fluorescein (Haag-Streit, Koenitz, Switzerland) was moistened with a drop of unpreserved, sterile saline solution 0.9% from a single-dose ampule, and then touched to the inferior fornix with minimal stimulation. The tear film was observed under a wide beam, cobalt blue-filtered light. The time (seconds) between the last complete blink and the first emergence of randomly distributed dry spots was recorded. Three consecutive measurements per subject eye were made (both right and left eyes) and the average of the three BUT values was taken as the mean value. A BUT of ≤ 5 s was used as a cut-off value for dry eye^13–15^. Un-anesthetized Schirmer test was performed for the estimation of tear production. Standardized strips of filter paper (Alcon Laboratory, Fort Worth, Texas, USA) were inserted at the lower-lid margin at the junction of the middle and temporal third of both eyes taking care not to touch the cornea. Patients and healthy volunteers were instructed to gently close their eyelids and not to move their eyes for 5 min. The strip was then removed and the length of the wet portion was measured (mm/5 min). A Schirmer value of ≤ 10 mm in 5 min was recognized as abnormal. IOP was measured by Huvitz HNT-1P (Huvitz, Dongan-gu, Republic of Korea) noncontact tonometer; three measurements per eye were made and the average of the three IOP values was taken as the mean value. The Ocular Surface Disease Index (OSDI; Allergan Inc, Irvine, California), a 12-item self-administered questionnaire was recorded to estimate ocular surface symptoms, and grading of OSDI was performed according to previously published guidelines: normal ocular surface (0–12 points), mild (13–22 points), moderate (23–32 points), or severe (33–100 points) ocular surface disease^16^. SSc patients were stratified based on their OSDI score into 4 groups ranging from normal to severe.

### IVCM examinations and image analyses

All study subjects and controls underwent IVCM imaging of each corneal layer including the sub-basal nerve plexus of both eyes with Heidelberg Retina Tomograph III Rostock Cornea Module (HRT III RCM; Heidelberg Engineering GmbH, Heidelberg, Germany). Both eyes were anesthetized with topical anesthetic (tetracaine hydrochloride 0.4%) eye drop and ophthalmic gel (Vidisic Gel; Bausch&Lomb, Berlin, Germany) applied in a sterile polymethylmethacrylate cap (Tomo-Cap; Heidelberg Engineering GmbH), which was placed over the objective lens. Section scans were recorded from the epithelium to endothelium both for morphological and quantitative analysis.

Three good quality images of the basal epithelium, subbasal nerve plexus, anterior and posterior stroma and endothelium were selected. Epithelial, keratocyte, and endothelial cell densities were calculated using the instrument-based, semi-automated software as described previously^17^. The average of three measurements was used for further comparison. Three scans of the sub-basal nerve plexus were selected and analyzed using ACCMetrics software V3 (University of Manchester, Manchester, UK)^18–23^ to calculate corneal nerve fiber density (NFD), the number of nerve fibers/mm^2^; nerve branch density (NBD), the number of primary branch points on the main nerve fibers/mm^2^; nerve fiber length (NFL), the total length of nerves mm/mm^2^; nerve fiber total branch density (NTBD), the total number of branch points/mm^2^, nerve fiber area (NFA), the total nerve fiber area mm^2^/mm^2^; nerve fiber width (NFW), and the average nerve fiber width mm/mm^2^.

All measurements were performed between 9:00 a.m. and 11:00 a.m. to eliminate the effect of diurnal variations. Measurements were performed in the same room with constant light, temperature, humidity, and airflow, to avoid any ocular surface stress. Ambient temperature was 21 °C and relative humidity was 60 ± 3%. The right eyes of healthy subjects and SSc patients were used for comparative analysis.

### Statistical analyses

Descriptive statistical results are reported as mean, standard deviation (SD), and 95% confidence interval (CI). Statistical analysis was performed using MedCalc Version 14.8.1 and IBM SPSS Statistics 25.0. The Kolmogorov–Smirnov test was used for normality. For pairwise comparison, Mann–Whitney unpaired test (between groups) and Wilcoxon test (within groups) was used for nonparametric data. For bivariate correlation analysis, Spearman correlation test was applied. A P value ≤ 0.05 was considered statistically significant.

### Ethical approval

The study protocol was approved by the local ethics committee (Regional and Institutional Research Ethical Committee of the University of Debrecen) and was in full compliance with Good Clinical Practices (GCP) and GDPR guidelines of the European Union, and the Declaration of Helsinki (1996).

### Informed consent

Informed consent was obtained from all individual participants included in the study.

## Results

65 eyes of 33 patients with SSc (5 males and 28 females, mean age: 67.74 ± 9.39 years, between 46 and 85 years of age) and 30 eyes of 30 age-matched healthy subjects (15 males and 15 females, mean age: 64.55 ± 9.48 years, between 46 and 85 years of age) were studied (P = 0.169). Mean disease duration was 15.21 ± 8.04 years (between 2 and 29 years). Clinical characteristics of patients with SSc are detailed in Table 1.

**Table 1:** Clinical characteristics of patients with systemic sclerosis (SSc) in mean ± standard deviation (95% confidence interval). OSDI: ocular surface disease index; BUT: tear film breakup time.

	SSc patients	
	Right eye	Left eye
Age (years)	67.74 ± 9.39 (64.30–71.19)	
Gender	5 males, 28 females	
Disease duration (years)	15.21 ± 8.04 (12.25–18.16)	
Best corrected visual acuity	0.91 ± 0.16 (0.84–0.98)	0.89 ± 0.18 (0.81–0.97)
Intraocular pressure (mmHg)	14.31 ± 3.32 (13.05–15.57)	14.00 ± 3.10 (12.82–15.18)
OSDI score	33.27 ± 30.11 (22.42–44.13)	
Schirmer test (mm)	6.78 ± 5.82 (4.68–8.87)	6.25 ± 5.73 (4.19–8.31)
BUT (sec)	4.82 ± 3.15 (3.70–5.93)	5.09 ± 3.41 (3.88–6.30)

**Figure 1:**
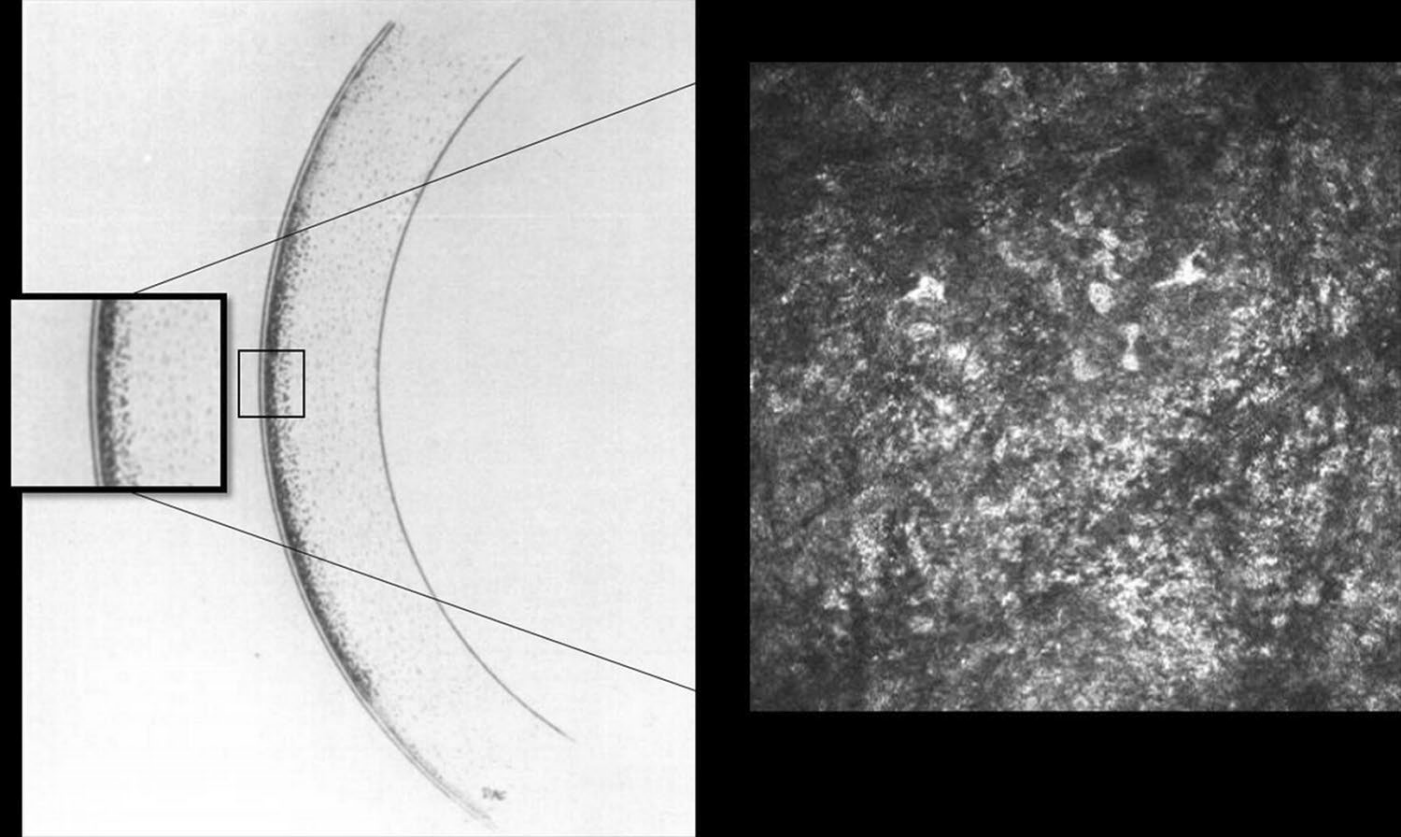
Drawing of Cockburn in 1984 demonstrating slit lamp narrow beam section of anterior stromal lesions in „systemic scleroderma” and a representative in vivo confocal microscopic photo of anterior stroma in a patient with systemic sclerosis. (Cockburn DM (1984) Corneal and Other Ocular Changes in Progressive Systemic Scleroderma. Clin Exp Optom 67:37–70. copyright Optometry Australia, reprinted by permission of Taylor & Francis Ltd, http://www.tandfonline.com on behalf of Optometry Australia.).

**Figure 2:**
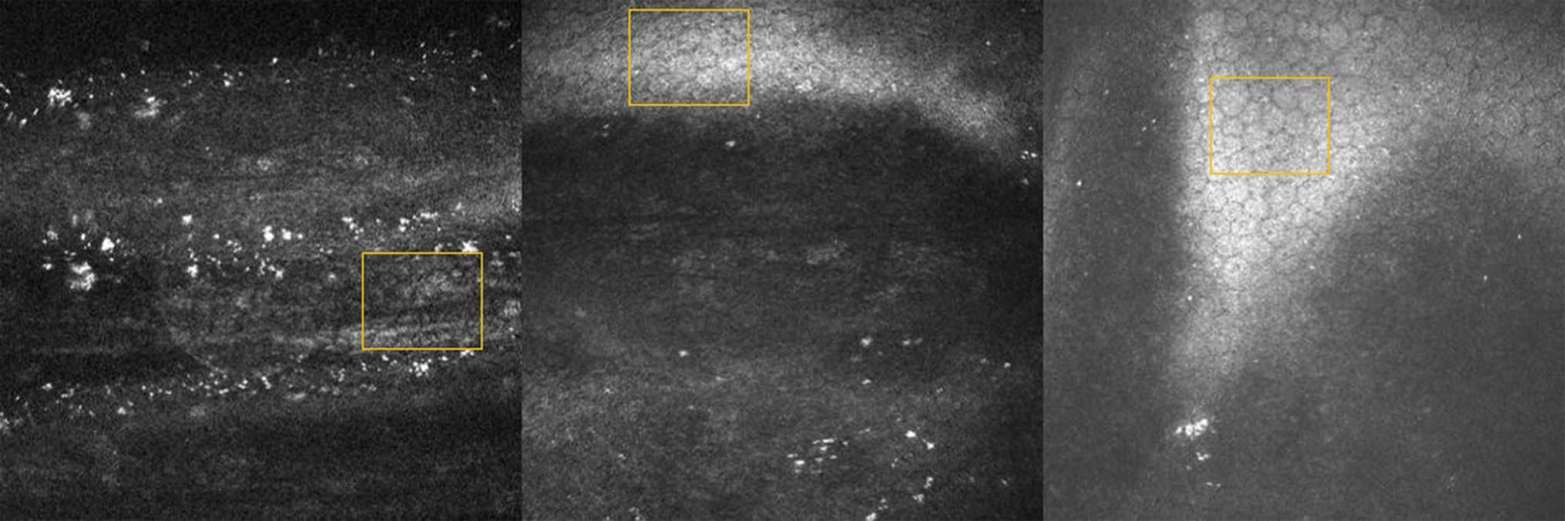
In vivo confocal microscopy photo of three systemic sclerosis patients (disease duration of 29, 23, 17 years respectively) with accumulation of punctate hyperreflective material in the level of Descemet’s membrane/endothelium. Note the normal endothelial cells in the yellow square.

**Figure 3:**
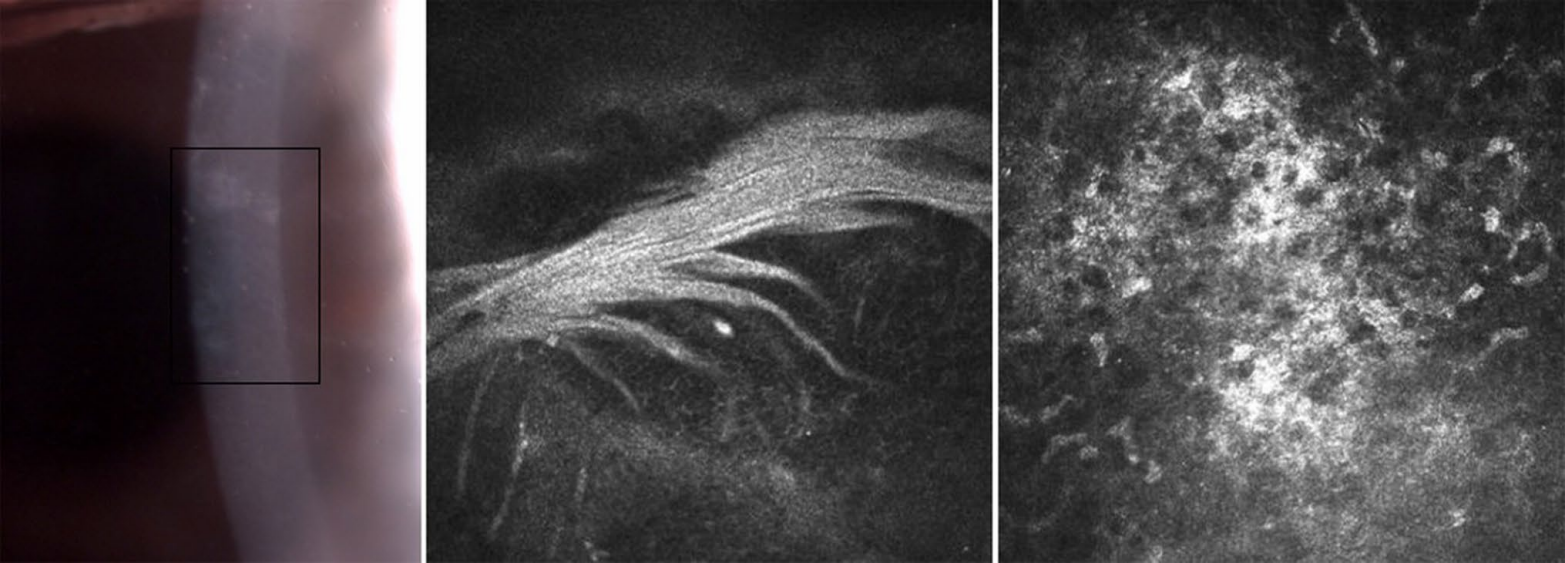
Slit lamp (left) and in vivo confocal microscopy photos of a systemic sclerosis patient with long standing disease. Note the whorl-like hyperreflective, extracellular material in the basal epithelium (middle) and activated keratocytes with accumulated dot-like lesions in the anterior stroma (right).

**Figure 4:**
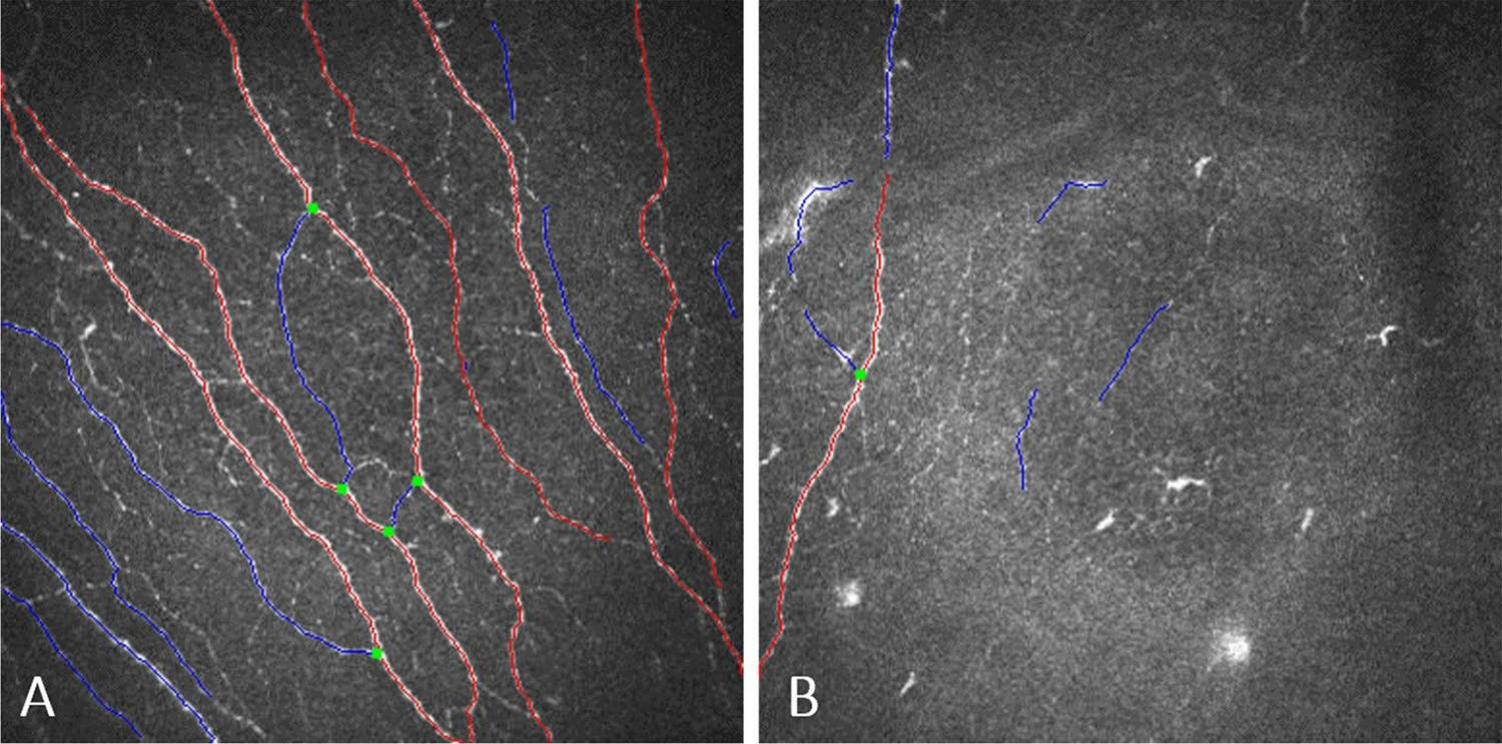
Annotated image of the subbasal nerve plexus using ACCMetrics software V3 (red, fiber; blue, branch; green, branch point) (https://weillcornell.az1.qualtrics.com/jfe/form/SV_6o2ji0suM4jQinb). (**a**) normal nerve fiber morphology of a healthy individual. (**b**) Decreased nerve fiber density and altered morphology of a patient with systemic sclerosis for 29 years.

Compared to control basal epithelial, posterior stromal keratocyte and endothelial cell densities were lower, but not significantly so, in SSc patients (P = 0.156, P = 0.095, P = 0.391 respectively) (Table 2). Keratocyte cell density in the anterior stroma was significantly lower in patients with SSc compared to controls (P < 0.0001). In 7 patients with SSc no keratocyte nuclei were identified in the anterior stroma and in most patients scattered hyperreflective punctate material in the anterior stroma with only a few normal keratocyte nuclei were noted (Fig. 1). Three SSc patients showed accumulation of hyperreflective material in the posterior stroma. Similar dot-like lesions were seen in the level of Descemet’s membrane/endothelium (Fig. 2) in five systemic sclerosis patients. One SSc patient with 29 years of disease duration had whorl-like hyperreflective, extracellular material in the basal epithelium with some activated keratocytes and accumulated punctate lesions in the anterior stroma (Fig. 3). None of the above-mentioned alterations was observed in the healthy control group. There was no significant difference in any of the corneal cell density parameters between right and left eye of SSc patients (P = 0.052–0.748). There was no significant correlation between the duration of systemic sclerosis and any of the corneal cell density values (P > 0.05). Significantly lower subbasal nerve fiber parameters were found in patients with SSc compared to those of the healthy control subjects (Fig. 4, Table 2). There was a significant correlation between NFW (r = 0.311, P = 0.009) and an inverse correlation between NBD (r = −0.280, P = 0.019) and NFL (r = −0.288, P = 0.016) with disease duration.

**Table 2:** Corneal microstructural alterations and subbasal nerve plexus morphology in SSc patients compared to healthy subjects in mean ± standard deviation (95% confidence interval). SSc: systemic sclerosis; *Mann–Whitney unpaired test.

	Healthy subjects	SSc patients	P*
Central corneal thickness (µm)	517.83 ± 50.31 (505.47–530.20)	513.75 ± 54.87 (499.20–528.31)	0.124
Epithelial cell density (cells/mm^2^)	7629.67 ± 886.95 (7433.55–7825.79)	7407.28 ± 999.13 (7201.511–7613.05)	0.156
Anterior stromal keratocyte cell density (cells/mm^2^)	229.58 ± 36.10 (221.60–237.56)	187.93 ± 79.29 (170.83–205.03)	< 0.0001
Posterior stromal keratocyte cell density (cells/mm^2^)	238.97 ± 29.95 (232.08–245.86)	230.67 ± 45.34 (220.09–241.25)	0.095
Endothelial cell density (cells/mm^2^)	2871.02 ± 298.89 (2797.54–2944.49)	2733.93 ± 541.46 (2590.26–2877.60)	0.391
Nerve Fibre Density (No/mm^2^)	16.63 ± 8.78 (14.91–18.34)	10.78 ± 14.42 (7.49–13.91)	< 0.0001
Nerve Branch Density (No/mm^2^)	18.62 ± 16.99 (15.31–21.95)	10.70 ± 7.59 (8.99–17.09)	0.0002
Nerve Fibre Length (mm/mm^2^)	11.57 ± 3.88 (10.81–12.33)	9.02 ± 3.92 (8.15–9.90)	< 0.0001
Nerve Fibre Total Branch Density (No/mm^2^)	32.34 ± 23.60 (27.72–36.95)	23.44 ± 21.96 (18.55–28.32)	0.005
Nerve Fibre Area (mm^2^/mm^2^)	0.0054 ± 0.0021 (0.0049–0.0058)	0.0040 ± 0.0016 (0.0037–0.0044)	< 0.0001
Nerve Fibre Width (mm/mm^2^)	0.021 ± 0.0016 (0.0211–0.0215)	0.022 ± 0.0022 (0.0214–0.0224)	0.023

There was no statistically significant difference between gender in any of the measured parameters (P value was ranging between 0.075 and 0.962). BUT was ≤ 5 s in 47 eyes (72%), Schirmer value was ≤ 10 mm in 50 eyes (77%) and was ≤ 5 mm in 40 eyes (62%). The OSDI score showed normal value in 12 patients (36%), it indicated mild ocular surface disease in 3 patients (9%), moderate disease in 2 patients (6%) and severe ocular surface damage in 16 patients (49%). There was no significant difference in central corneal thickness (P = 0.124) between patients with systemic sclerosis and healthy subjects (Table 2).

After stratifying patients based on their OSDI score, no statistically significant difference was obtained between the four groups in any of the nerve fiber parameters (P = 0.103–0.969).

## Discussion

Ocular involvement is not uncommon in autoimmune or connective tissue diseases. Some SSc patients with ocular complications have eye symptoms in parallel with, others prior to the diagnosis, while in some cases symptoms are viewed as unrelated to the systemic disease. An undiagnosed or improperly treated systemic disease can lead to severe ocular morbidity. As a consequence, information about the ocular findings of autoimmune diseases is valuable for rheumatologists, immunologists, and ophthalmologists. In the present study, corneal ultrastructural alterations were examined in patients with SSc including cellular, extracellular matrix (ECM) changes and subbasal nerve plexus morphology.

SSc is a complex connective tissue disorder with altered innate and adaptive immune responses leading to excessive collagen and ECM accumulation in different organs^24^. As part of these immune responses, a cascade of cytokines and chemokines are released and induce fibroblast proliferation^2,25^. The first mention of corneal structure anomaly in SSc was described by Cockburn in 1984. He reported punctate opacities beneath Bowman’s membrane and in the anterior stroma^26^. In the healthy human corneal stroma, keratocytes are quiescent, but can be activated and transdifferentiated into myofibroblasts which can actively produce ECM and fibrosis^27^. Fibrosis in the corneal stroma could be part of a physiological tissue repair process under abnormal conditions such as in autoimmune diseases. In SSc, both activated keratocytes and inflammatory cells are assumed to secrete signaling molecules and growth factors which may result in overproduction of ECM components. In our SSc group, 7 individuals did not show any normal keratocyte nuclei in the anterior stroma, however, we observed activated keratocytes and accumulated hyperreflective punctate material in the superficial stroma. In five SSc patients, similar lesions were detected in the level of Descemet’s/endothelium. Impaired extracellular matrix degradation by matrix metalloproteinases has been suggested to play an important role in the pathogenesis of SSc^28^. The imbalance between the synthesis and degradation processes might lead to the accumulation of degraded material in the corneal layers. The circulating or urinary levels of collagen molecules or fragments have been investigated as biomarkers that might reflect the disease activity^29^.

The best corrected visual acuity was not impaired in most of our SSc cases suggesting these ultrastructural changes may not have an impact on the visual performance. Corneal transparency is determined by the diameter of and distance between collagen fibers in the stroma. Keratocyte nuclei may be a significant source of light scattering; however, their refractive properties are similar to the surrounding stroma when they are quiescent. We observed lower keratocyte density in our SSc patients, but in some cases we detected keratocyte activation which should have a different refractive index. Light scattering is a complex process as it depends on the size, shape and refractive properties of the optical media relative to the surrounding structure. It is also related to the space between structures of different refractive indices and the magnitude of difference in refractive index of neighboring structures^30^.

Normal corneal stroma is mostly composed of type I collagen with smaller amounts of types III and V which are extensively affected in systemic sclerosis^31–33^. We detected slightly lower central corneal pachymetry values in patients with SSc when compared to healthy subjects. Corneal volume was not significantly different between subjects and controls. This finding is in accordance with previous reports; Sahin et al. also reported decreased corneal thickness in patients with systemic sclerosis^34^. However, cellular elements of corneal stroma comprise only 2–3% of the stromal volume^35^. In our SSc patients, we did not observe any significant difference in epithelial and endothelial cell density, but the anterior stromal keratocyte number was significantly lower when compared to controls. The above-mentioned changes in corneal morphology did not correlate with the duration of SSc; only some nerve fiber morphology parameters showed significant correlation with disease duration.

The overall understanding of corneal manifestations from immunological and rheumatic inflammatory disorders has continued to expand over the past few years. IVCM techniques accomplish a wide depth-of-focus to observe in vivo microstructure of the cornea^36,37^. Diverse lesions, including definite changes in epithelial cell morphology^38^, keratocytes activation^39^, as well as irregular morphologic changes, increased number of subbasal nerve plexuses or tortuosity^40,41^ have been demonstrated in corneas of patients with dry eye disease by IVCM. IVCM studies also reported corneal ultrastructure changes following immune-mediated inflammatory diseases^42,43^. In primary Sjögren’s syndrome (pSS) superficial epithelial cell density was significantly reduced compared with no-SS dry eye; moreover, keratocyte activation and subbasal nerve irregularities were also dominant^44^. These previously reported alterations are consistent with our findings. Based on the dry eye tests and OSDI score, SSc patients in our study suffered from mild to severe ocular surface disease. However, after stratifying patients based on their dry eye symptoms, there were no significant difference in the nerve fiber morphology between the groups.

New imaging technologies, such as IVCM, have expanded our knowledge about corneal morphology^45^. Using an automated analysis software we quantified the subbasal nerve morphological alterations in systemic sclerosis. We detected significant damage to the nerve fibers in our SSc group. This is in accordance with other studies reporting considerable prevalence of peripheral neuropathy in patients with systemic sclerosis^46^.

A healthy ocular surface environment is essential for good quality vision. To the best of our knowledge, this is the first study investigating corneal morphological alterations in vivo in patients with systemic sclerosis. We confirmed and documented certain corneal ultrastructural changes in SSc which have been described by Cockburn in 1984^26^. Severe ocular surface disease was also observed in SSc patients with significant impairment of the subbasal nerve plexus morphology resembling peripheral neuropathy. Moreover, a significant association of nerve fiber changes with disease duration was observed. Our results support the conclusion that IVCM offers an easy method of routine screening for neuropathy in SSc patients which may reduce further morbidity of these patients.
